# Inpatient Telehealth Experience of Patients With Limited English Proficiency: Cross-sectional Survey and Semistructured Interview Study

**DOI:** 10.2196/34354

**Published:** 2022-04-19

**Authors:** Lily Payvandi, Chase Parsons, Fabienne C Bourgeois, Jonathan D Hron

**Affiliations:** 1 Division of General Pediatrics Department of Pediatrics Boston Children's Hospital Boston, MA United States; 2 Department of Pediatrics Harvard Medical School Boston, MA United States

**Keywords:** telemedicine, telehealth, inpatient, social determinants of health, limited English proficiency, health-related social needs, Spanish, English as a second language, English proficiency

## Abstract

**Background:**

Patients with limited English proficiency (LEP) are at a higher risk of poor health outcomes and are less likely to use telehealth than English-speaking patients. To date, there is no formal evaluation of inpatient (IP) telehealth user experience of patients and their families by language preference during visits with their clinicians.

**Objective:**

This study aims to compare the experiences of English- and Spanish-speaking patients and their families using IP telehealth, as well as to evaluate the experience of Spanish interpreters providing services through IP telehealth.

**Methods:**

We prospectively administered a survey to English- and Spanish-speaking patients and their families who used IP telehealth from October 1, 2020, to March 31, 2021. We performed semistructured phone interviews of hospital-based Spanish interpreters who provided services through IP telehealth.

**Results:**

A total of 661 surveys were administered, with completion rates of 18% (112/621) in English and 62% (25/40) in Spanish. On a 10-point scale, the overall satisfaction of Spanish speakers (median 10, IQR 10-10) was higher than that of English speakers (median 9, IQR 8-10; *P*=.001). Both English- and Spanish-speaking patients used IP telehealth for visits with their primary IP care team, subspecialty consultants, and other clinicians. Hospital tablets were used more often than personal devices, and only English-speaking patients used personal laptops. Patients and their families encountered challenges with log-in, team coordination with multiple users, and equipment availability. Interpreters encountered challenges with audio and video quality, communication, safety, and Wi-Fi access.

**Conclusions:**

Both English- and Spanish-speaking patients reported high satisfaction using IP telehealth across multiple disciplines despite the workflow challenges identified by interpreters. Significant investment is needed to provide robust infrastructure to support use by all patients, especially the integration of multiple users to provide interpreter services for patients with LEP.

## Introduction

Health care delivery required redesign during the COVID-19 pandemic. Given the limited availability of personal protective equipment, several aspects of health care were transformed to safely deliver high-quality care from a distance [1,2]. Critical to this redesign was the expansion of telehealth. Although telehealth use in inpatient (IP) settings is not new [3-7], it previously had poor market penetration due to strict regulation and low rates of reimbursement that varied by state [8,9]. IP telehealth expansion during the pandemic was used to support infection control practices, including minimizing personal protective equipment use and the surge of patient demand on hospital systems [10-12].

Beyond infection control, there were simultaneous, ongoing public health crises illuminated during the COVID-19 pandemic, which included health inequity [13]. Equity is a core component of health care access and quality and should be explicitly considered during system design and quality improvement [14]. The legal expectation to provide language access across all federal programs, including health care, originated from the Civil Rights Act and has been expanded under Section 1557 of the Affordable Care Act [15,16]. Successful telehealth implementation is critical to addressing health inequity, which includes linguistic barriers. Patients with limited English proficiency (LEP) have increased risks of poor health outcomes, adverse hospital events, and 30-day emergency department revisits compared to English-proficient patients [17]. Although non–English language preference is associated with higher interest in ambulatory video visits, having LEP is associated with a lower likelihood of ambulatory or urgent care video visits [18-20].

We previously described the rapid implementation of an IP telehealth program to facilitate IP care during the COVID-19 pandemic [21]. Prior evaluation of patient satisfaction with IP telehealth has shown high satisfaction with COVID-19 medical units, rehabilitation, and psychiatric partial hospitalization [22-24]. However, there has not yet been a formal evaluation of IP telehealth user experience by language preference. The experience of users with LEP is dependent on the seamless integration of interpreters; thus, to support this population, we also need to support the interpreter workflow. Our objective is to compare the experiences of English- and Spanish-speaking patients and their families using IP telehealth, as well as to evaluate the experience of Spanish interpreters providing services through IP telehealth.

## Methods

### Study Setting and Data Collection

This prospective survey study was conducted at Boston Children’s Hospital, an academic quaternary care pediatric facility in Boston, Massachusetts. We conducted a mixed methods study of quantitative patient survey data and qualitative interpreter interview data.

### Evaluation of the Experience of Patients and Their Families Using IP Telehealth

An 8-question survey (Multimedia Appendix 1) was developed via REDCap (REDCap Consortium) and modified based on the institution’s outpatient virtual visit survey. The survey was reviewed and edited by the health literacy team to ensure accessible language use. The survey was translated into Spanish by Boston Children’s Hospital Interpreter Services.

Patients and their families who used IP telehealth from October 1, 2020, to March 31, 2021, were identified by crossreferencing reports from the videoconferencing software with IP encounters in the enterprise data warehouse. We further filtered the report by selecting all patients with English or Spanish as their primary language. *Language* in this study is defined as the preferred language listed in the patient’s electronic health record. Emails and phone numbers were used for survey distribution. Use of IP telehealth in dialysis, operating rooms, and radiological imaging were excluded as these visit types could not be linked to specific patient encounters. Patients with email listings were sent the survey in their listed language. Patients without email listings were called via phone, and the survey was administered in their listed language by author LP or a trained Spanish interpreter. Due to an initially low Spanish survey response rate and fewer email listings, starting from December 1, 2020, all surveys were administered by phone to patients with Spanish listed as their preferred language. Survey results of de-identified patients were downloaded from REDCap.

### Evaluation of the Experience of Interpreters Providing Services Through IP Telehealth

The hospital has a dedicated interpreter services department with 23 staff members available either in person or by phone or videoconferencing, in addition to external interpreters via contracted agencies. During the COVID-19 pandemic, interpreters initially transitioned to remote work exclusively, but during the study period, they were available in person and remotely. Clinicians typically contacted interpreters by paging to arrange a meeting time. With the launch of an IP telehealth initiative, clinicians were instructed to provide a telehealth meeting link to the interpreter when requesting services. There is also a virtual interpreter system provided by a third-party vendor via tablets on wheels in each hospital unit; however, this system was not integrated with IP telehealth at the time of this study. We conducted semistructured phone interviews of 5 hospital-based Spanish interpreters who provided services through IP telehealth to assess their experience with the program and how it may be improved. The semistructured interview guide consisted of the following questions: (1) What issues did you experience during the IP telehealth session? (2) What do you like about IP telehealth? and (3) What are the opportunities for IP telehealth improvement? Responses were reported to include a representative sample and were categorized inductively.

### Ethical Considerations

This project was part of a larger quality improvement effort at Boston Children’s Hospital and was thus deemed exempt from Institutional Review Board review by the Department of Pediatrics Performance Excellence Group.

### Statistical Analysis

Medians and IQRs were calculated for continuous variables. A *P* value of .05 was considered statistically significant. Wilcoxon rank sum test with continuity correction was performed.

## Results

### Evaluation of the Experience of Patients and Their Families Using IP Telehealth

Of the 8422 unique patients admitted to the hospital during the study period, 505 (6%) had Spanish listed as their preferred language. Of these 505 unique Spanish-speaking patients, 40 (7.9%) used IP telehealth during the study period. A total of 661 eligible patients were identified and administered the survey, with completion rates of 18% (112/621) in English and 62% (25/40) in Spanish. On a 10-point scale, the overall satisfaction of Spanish speakers (median 10, IQR 10-10) was higher than that of English speakers (median 9, IQR 8-10; *P*=.001). On a 10-point scale, the median scores for how well they felt their questions were answered during their visit were equal: 10 (IQR 10-10) for Spanish speakers and 10 (IQR 9-10) for English speakers (*P*=.03). Both English- and Spanish-speaking patients reported IP telehealth visits with their primary IP care team, subspecialty consultants, and other clinicians (Table 1).

Hospital tablets were used more often than personal devices, and notably, only English-speaking patients used personal laptops (Table 2).

Of patient and family respondents with Spanish listed as their preferred language, 80% (20/25) reported using an interpreter during their visit. Feedback on how to make the system easier to use was most frequently regarding log-in, team coordination with multiple users including interpreters, and equipment availability (Table 3). We coded the responses into categories.

**Table 1:** Comparison of inpatient telehealth visit type by language.

Telehealth visit type	English-speaking respondents^a^ (n=112), n (%)	Spanish-speaking respondents^a^ (n=25), n (%)
Primary inpatient care team	54 (48)	12 (48)
Specialist consult	50 (45)	12 (48)
Other	48^b^ (43)	9^c^ (36)

^a^The sum of each column is greater than the number of respondents because the survey question permitted multiselect answers. The denominator for each cell is the number of respondents.

^b^Other telehealth visit types for English-speaking respondents included behavioral health therapy, child life, dietician, music therapy, nasogastric pump instructions, parent calling patient, patient calling provider from outside facility, patient unsure of type, pet therapy, and social work.

^c^Other telehealth visit types for Spanish-speaking respondents included dietician, music therapy, patient unsure of type, pet therapy, and social work.

**Table 2:** Comparison of inpatient telehealth device type by language.

Device type	English-speaking respondents (n=112), n (%)	Spanish-speaking respondents (n=25), n (%)	All respondents (N=137), n (%)
Hospital tablet	50 (45)	14 (56)	64 (47)
Personal phone or tablet	36 (32)	11 (44)	47 (34)
Personal laptop	26 (23)	0 (0)	26 (19)

**Table 3:** Categorized comment responses (N=52) to the survey question “How can we make the system easier to use?”

Categories for improvement	Responses, n (%)
Log-in	8 (15)
Multiple users/team coordination	5 (10)
Equipment availability	3 (6)
Privacy	3 (6)
Poor internet connection	2 (4)
Patient engagement	2 (4)
“Unreliable” interpreter	1 (2)
Audio quality	1 (2)
Advertising	1 (2)
Positive comments	26 (50)

### Evaluation of the Experience of Interpreters Providing Services Through IP Telehealth

Feedback categories derived from phone interviews with Spanish interpreters include audio and video quality, communication, safety, and Wi-Fi access (Table 4).

**Table 4:** Interpreter phone interview feedback.

Category	Sentiment	Illustrative quote
General	Positive	Thank you for caring about our Latino population!
Safety	Positive	This helped me and my family stay safe from the virus. I have children at home and was worried about working in person and spreading COVID to them.
Audio quality	Constructive	It is challenging to hear the team when they are in full PPE [personal protective equipment]. Make sure the iPad is close to the patient, volume is high, and it is clear who is taking turn to speak. Otherwise it is overwhelming.
Communication	Constructive	Our Latino population is more responsive with texts than emails. Can we send the invite link through a text instead of an email? Physical therapy was difficult because we could not touch the patient to instruct them to turn around.
Video quality	Constructive	It is tough to visualize the patient and family at the same time. We need more family education on pointing the camera at the correct angle.
Wi-Fi access	Constructive	Most patients [who I interpret for] do not have Wi-Fi at home so they use data on their cell phones. We should help them use hospital Wi-Fi to download the application on their phones while inpatient. This will help them with their follow-up outpatient televisits.

## Discussion

### Principal Findings

We found that the patients in this sample had high satisfaction with IP telehealth, but Spanish-speaking patients’ median satisfaction scores were higher than those of English speakers despite the workflow challenges identified by interpreters. Broad use cases of IP telehealth were present for both language groups, including visits with the primary IP care team, specialty consultants, social workers, and dieticians. Overall, hospital tablets were used more than personal devices, especially by Spanish-speaking patients. This finding suggests the need for further investment in dedicated hospital devices. Survey respondents provided useful feedback that was shared in departmental educational sessions and can inform future improvements (eg, regarding session log-in and audio and video quality with multiple team members).

Notably, interpreters expressed appreciation for the opportunity to deliver care from a distance during the COVID-19 pandemic. This finding highlights the value of IP telehealth as a tool to ensure staff safety from infectious diseases.

### Comparison With Prior Work

Previous literature on IP telehealth implementation has described a broad range of use cases and patient satisfaction that is comparable to in-person care [22-26]. Our study adds to this literature by specifically addressing the experience of patients and their families who have LEP. The positive experience reported by Spanish speakers in our survey aligns with prior work in ambulatory settings that shows high interest in telehealth among non–English speakers [20].

Spanish-speaking patients primarily using hospital tablets and not personal laptops emphasizes the importance of investing in IP telehealth infrastructure for patients with LEP [27]. In one paper, narratives of patients with LEP who experience social isolation while hospitalized highlight the importance of careful tablet technology implementation to address communication barriers [28]. Other hospital systems describe the use of Amazon Echo Show devices, laptops on wheels, and tablets on wheels as part of telehealth [25,26]. Carts with the ability to pan and tilt, hands-free voice-activated command devices, and supplementary speakers may also help address the audio and video quality challenges noted by survey respondents.

Similar to previous findings, we found that work-arounds for integrating multiple users to include interpreters compromised audio and video quality [25]. Literature has shown better communication outcomes for interpreters connected by video than by phone, further supporting the need for robust infrastructure, including integrated interpreter services, to support IP telehealth initiatives [29].

### Limitations

This study has limitations, particularly the switch to phone-administered surveys for Spanish-speaking patients due to a low response rate and fewer email listings than English-speaking patients. The use of phone surveys may introduce acquiescence or conformity bias, which may explain the higher median satisfaction score among Spanish-speaking respondents than English-speaking respondents. Satisfaction may also be inflated due to a perceived lack of anonymity or a fear of disapproval from health care professionals [30,31]. In addition, language preference as documented in the electronic health record may not accurately identify patients with LEP. This could lead to miscategorization, affecting both our survey response rate and survey results. Our mixed methods approach meant that the interpreters—a small cohort—were interviewed, whereas the larger cohort of patients received the structured survey. Survey data have limitations that include inflexibility and lack of depth, which we attempted to address by including textboxes for open-ended questions. Finally, our findings may not apply to patients who speak languages other than English and Spanish, although this institution supports 15 languages through in-house interpreters and more than 100 languages through externally contracted interpreter services.

### Conclusions

Both English- and Spanish-speaking patients reported high satisfaction using IP telehealth across multiple disciplines despite the workflow challenges identified by interpreters. Significant investment is needed to provide robust infrastructure to support use by all patients, especially the integration of multiple users to provide interpreter services for patients with LEP.

## Appendix

Multimedia Appendix 1 Patient survey in English and Spanish.

## Abbreviations

IP: inpatient
LEP: limited English proficiency
