# Supplementary material for: Inpatient Telehealth Experience of Patients With Limited English Proficiency: Cross-sectional Survey and Semistructured Interview Study
Source: JMIR Form Res. 2022 Apr 19;6(4):e34354. doi: 10.2196/34354 (PMC9066319; doi:10.2196/34354)
Supplement: Multimedia Appendix 1 [file formative_v6i4e34354_app1.docx]

**Appendix Table**

Survey Questions in English and Spanish.

| Survey Questions in English | Survey Questions in Spanish |
| --- | --- |
| Do you prefer to answer the survey in English or in Spanish? | ¿Prefiere usted contester este cuestionario en inglés o en español? |
| On a scale from 1 to 10 (where 1 is the worst possible experience and 10 is the best possible experience), how would you rate the virtual visit experience overall? | En una escala de 1 a 10 (donde 1 es la peor experiencia posible y 10 es la mejor experiencia posible) ¿ Cómo calificaría usted su experiencia con la cita virtual en general? |
| On a scale from 1 to 10 (where 1 is not well at all and 10 is very well), how well were you able to get answers to your questions during your visit? | En una escala de 1 a 10 (donde 1 es para nada bien y 10 es muy bien) ¿Qué tan bien se le dio respuestas a sus preguntas durante la cita? |
| Was a language interpreter used during your visit? | ¿Tuvo un intérprete durante su cita? |
| Please select the type of virtual visit you’re describing. | Por favor seleccione el tipo de cita virtual que está describiendo. |
| Which device type did you use to conduct the visit? | ¿Qué tipo de dispositivo utilizó usted para la cita? |
| Did you experience any issues during the virtual visit? | ¿Tuvo alg**ún problema durante la cita virtual?** |
| How can we make the system easier to use? | ¿Cómo podríamos mejorar el sistema para que sea más fácil de usar? |
